# Countering opioid-induced respiratory depression by non-opioids that are respiratory stimulants

**DOI:** 10.12688/f1000research.21738.1

**Published:** 2020-02-07

**Authors:** Mohammad Zafar Imam, Andy Kuo, Maree T Smith

**Affiliations:** 1 School of Biomedical Sciences, Faculty of Medicine, The University of Queensland, Brisbane, Queensland, Australia

**Keywords:** opioid, respiratory depression, respiratory stimulant, ampakine, allosteric modulator, NMDA receptor antagonist, 5-HT1a, 5-HT3

## Abstract

Strong opioid analgesics are the mainstay of therapy for the relief of moderate to severe acute nociceptive pain that may occur post-operatively or following major trauma, as well as for the management of chronic cancer-related pain. Opioid-related adverse effects include nausea and vomiting, sedation, respiratory depression, constipation, tolerance, and addiction/abuse liability. Of these, respiratory depression is of the most concern to clinicians owing to the potential for fatal consequences. In the broader community, opioid overdose due to either prescription or illicit opioids or co-administration with central nervous system depressants may evoke respiratory depression. To address this problem, there is ongoing interest in the identification of non-opioid respiratory stimulants to reverse opioid-induced respiratory depression but without reversing opioid analgesia. Promising compound classes evaluated to date include those that act on a diverse array of receptors including 5-hydroxytryptamine, D _1_-dopamine, α-amino-3-hydroxy-5-methyl-4-isoxazolepropionic acid (AMPA), N-methyl-D-aspartate (NMDA) receptor antagonists, and nicotinic acetylcholine as well as phosphodiesterase inhibitors and molecules that act on potassium channels on oxygen-sensing cells in the carotid body. The aim of this article is to review recent advances in the development potential of these compounds for countering opioid-induced respiratory depression.

## Introduction

Although the incidence of opioid-induced respiratory depression in the post-operative setting is low, it is of major concern to clinicians because of the potential for fatal consequences when clinical monitoring is inadequate. Of additional concern is the large increase in opioid-related deaths over the past decade due to respiratory depression, particularly in overdose and in individuals consuming other central nervous system depressants such as sedatives and alcohol ^1^. The opioids may have been prescribed for the management of chronic pain or they may have been obtained through diversion of prescribed opioids or by illicit means. Opioid-related deaths due to respiratory depression have risen in parallel with the marked increase in opioid consumption, particularly in the United States of America, over this period ^2^. Disturbingly, chronic opioid use accounts for an estimated 24% of central sleep apnea that can go unnoticed and be fatal without appropriate intervention ^3^. Apart from strategies aimed at risk mitigation by reducing clinical opioid administration, drug discovery programs have been aimed at discovering a new generation of opioids that retain potent analgesic activity but with less respiratory depression ^4– 6^. Another strategy, which is the subject of this review, is to identify respiratory stimulant molecules for potential co-administration with an opioid analgesic to counter opioid-related respiratory depression whilst sparing opioid analgesia.

## Recent advances in countering opioid-induced respiratory depression

Classes of molecules showing promising preclinical and/or clinical results to date include ampakines, 5-hydroxytryptamine (5-HT) receptor agonists, phosphodiesterase-4 inhibitors, D _1_-dopamine receptor agonists, nicotinic acetylcholine receptor agonists, acetylcholine esterase inhibitors, bradykinin receptor antagonists, N-methyl-D-aspartate (NMDA) receptor antagonists, protein kinase A inhibitors, G-protein-gated inwardly rectifying potassium channel (GIRK) blockers, α _2_-adrenoceptor antagonists, and chemoreceptor stimulants (see summary in Table 1). For a more detailed discussion, see the excellent review by Dahan and colleagues ^2^. Herein, we have focused only on the most recent research on these experimental respiratory stimulants.

**Table 1.** Summary of non-opioid molecules assessed for their ability to counter opioid-induced respiratory depression.

Pharmacological class	Molecule	Dose, route	Receptor/target interaction	Co-administered opioid (dose)	Species (strain/sex)	Effect	Reference
*Ampakines*	CX717	1,500 mg, oral	AMPA	Alfentanil (100 ng/ml plasma concentration)	Human (males)	↑ Respiratory frequency; ↑ hemoglobin oxygenation; less decrease of slope of the linear relationship between expiratory volume/minute and CO _2_ concentration in expired air (in hypercapnic challenge)	18
		15 mg/kg, i.v.	AMPA	Fentanyl (60 µg/kg, i.v.)	Rat (SD)	↑ Respiratory frequency; ↑ oxygen saturation	19
		15 mg/kg, i.v.	AMPA	Fentanyl (60 µg/kg, i.v.)	Rat (SD)	↑ Respiratory frequency and amplitude	20
	CX546	16 mg/kg, i.p.	AMPA	Fentanyl	Rat (SD)	↑ Respiratory frequency; ↑ burst amplitude; no effect on behavior or arousal state	21
		15 mg/kg, i.p.	AMPA	Morphine (10 mg/kg, i.p.)	Rat (SD)	↑ Respiratory rate; ↑ tidal volume; ↑ minute ventilation	22
	CX1942		AMPA	Etorphine (0.1 mg/kg, i.v.)	Boer goat ( *Capra* *hircus*)	↑ Tidal volume; ↑ ventilation; ↑ PaO _2_; ↑ SaO _2_; ↓ PaCO _2_	12
	LCX001	10 mg/kg, i.v.	AMPA	Fentanyl (120 μg/kg, s.c.)	Rat (SD)	↑ Respiratory rate; ↑ minute ventilation	9
	XD-8-17C	1–30 mg/kg, i.v.	AMPA	TH-030418 (acute death – 15 mg/kg, s.c.; respiration – 20 µg/kg, i.v.)	Mouse (KM), rat (SD)	Protection against acute opioid-induced death; reversal of depression of respiratory parameters (respiratory frequency, minute ventilation, pO _2_, sO _2_) to normal; no effect on morphine antinociception	23
	Tianeptine	2 and 10 mg/kg, i.p.	AMPA	Morphine (10 mg/kg, i.p.)	Rat (SD)	↑ Respiratory rate; ↑ tidal volume; ↑ minute ventilation	22
*5-HT agonists*	Buspirone	50 µg/kg, i.v.	5-HT _1A_	Morphine (21.3 ± 2.1 mg/kg, i.v.)	Rat (SD)	Counteracted morphine-induced apnea	24
	Repinotan	10 and 20 μg/kg, i.v.	5-HT _1A_	Remifentanil (2.5 µg/kg, i.v.)	Rat (SD)	↑ Minute ventilation	25
	Befiradol	0.2 mg/kg	5-HT _1A_	Fentanyl (60 μg/kg, i.v.)	Rat (SD)	↑ Respiratory frequency; ↑ tidal volume; ↑ minute ventilation	26
	BIMU8	1–2 mg/kg, systemic	5-HT _4A_	Fentanyl (10–15 μg/kg, systemic)	Rat (SD)	↑ Respiratory minute volume	27
	8-OH-DPAT	0.5 mg/kg, i.v.	5-HT _1A_ and 5-HT _7_	Etorphine hydrochloride (0.06 mg/kg, i.m.)	Boer goat ( *Capra* *hircus*)	↓ Time to recumbency; ↑ respiratory rate; ↑ PaO _2_; ↓ PaCO _2_	28
	8-OH-DPAT	10 or 100 µg/kg	5-HT _1A_	Morphine (21.3 ± 2.1 mg/kg, i.v.)	Rat (SD)	Counteracted morphine-induced apnea	24
	Zacopride	0.5 mg/kg, i.v.	5-HT _4_	Etorphine hydrochloride (0.06 mg/kg, i.m.)	Boer goat ( *Capra* *hircus*)	↓ Time to recumbency; ↑ respiratory rate; ↑ PaO _2_; ↓ PaCO _2_	28
*Phosphodiesterase-* *4 inhibitors*	Caffeine	20 mg/kg, i.v.	PDE4	Morphine (0.4 mg/kg/ minute, i.v.)	Rat	↑ Inspiratory time; ↓ respiratory rate	29
		3 and 10 mg/kg, i.v.	PDE4	Morphine (1.0 mg/kg, i.v.)	Rat (WH)	Recovered prolongation and flattening effect on inspiratory discharge in the phrenic nerve by morphine	30
	Rolipram	0.1 and 0.3 mg/kg, i.v.	PDE4	Morphine (1.0 mg/kg, i.v.)	Rat (WH)	Recovered prolongation and flattening effect on inspiratory discharge in the phrenic nerve by morphine	30
*D1-dopamine* *receptor agonists*	6-Chloro-APB	0.5–3 mg/kg	D _1_	Fentanyl citrate (15–35 µg/kg)	Cat	Reversal of fentanyl-induced abolition of phrenic and vagus nerve respiratory discharges and firing of bulbar post-inspiratory neurons	31
	Dihydrexidine	0.5–2.0 mg/kg	D _1_	Fentanyl citrate (15–35 µg/kg)	Cat	Reversal of fentanyl-induced abolition of phrenic and vagus nerve respiratory discharges and firing of bulbar post-inspiratory neurons	31
	SKF-38393	1.5–3 mg/kg	D _1_	Fentanyl citrate (15–35 µg/kg)	Cat	Reversal of fentanyl-induced abolition of phrenic and vagus nerve respiratory discharges and firing of bulbar post-inspiratory neurons	31
*BK-channel blocker*	GAL021	Stepped drug infusion	Carotid body	Alfentanil (stepped drug infusion)	Human –healthy	↑ respiratory rate; ↑ tidal volume	32
	GAL021	(0.6, 1.5, and 6.0 mg/ml; 0.04, 0.1, and 0.4 mg/kg/minute)	Carotid body	Morphine (10 mg/kg, i.v.)	Rat (SD)	↑ Minute volume; ↑ tidal volume; ↑ PaO _2_; ↑ pH; ↓ PaCO _2_	33
		5-minute load of 0.2 or 0.1 mg/kg/minute i.v. + maintenance infusion 0.1 or 0.05 mg/kg/minute	Carotid body	Morphine (3–4 mg/kg, i.v.)	Cynomolgus monkeys	↓ End-tidal carbon dioxide (ET _CO2_)	33
Chemoreceptor stimulant	Almitrine	0.03, 0.1 mg/kg/ minute, i.v.	Peripheral chemoreceptors	Morphine (10 mg/kg, i.v.)	Rat (SD)	*Normoxia*: ↑ respiratory frequency; ↑ tidal volume; *Hypoxia*: ↓ respiratory frequency; ↑ tidal volume (0.03 mg/kg/ minute); ↓ tidal volume (0.1 mg/ kg/minute)	34
	Doxapram	1 mg/kg, i.v.	Carotid body	Etorphine (0.1 mg/kg, i.v.)	Boer goat ( *Capra* *hircus*)	↑ Respiratory frequency; ↑ ventilation; ↑ PaO _2_; ↑ SaO _2_; ↓ PaCO _2_	12
Nicotinic acetylcholine receptor agonist	Nicotine	0.6 mg/kg, s.c.	α4β2	Fentanyl (35 µg/kg, s.c.)	Rat (SD)	↑ respiratory frequency; ↑ tidal volume; ↑ minute ventilation;	10
	A85380	0.03 to 0.06 mg/kg, s.c.	α4β2	Fentanyl (35 µg/kg, s.c.)	Rat (SD)	↑ respiratory frequency; ↑ tidal volume; ↑ minute ventilation	10
N-methyl-D- aspartate receptor antagonist	Esketamine	0.57 mg/kg, i.v., cumulative	NMDA	Remifentanil (0.1–0.5 ng/ml, i.v.)	Human – healthy	Stimulatory effect on ventilatory CO _2_ sensitivity	35
Protein kinase A (PKA) inhibitor	H89	50 µg, i.c.v.	–	Fentanyl (60 µg/kg)	Rat (SD)	↑ respiratory frequency; ↑ inspiratory time; ↓ expiratory time	36
GIRK channel blocker	Tertiapin-Q	0.5–2 µg, i.c.v.	–	Fentanyl (60 µg/kg)	Rat (SD)	↑ respiratory frequency; ↑ inspiratory time	36
Alpha 2- adrenoceptor antagonist	SK&F 86466	1 and 5 mg/kg, i.v.	α _2_-adrenoceptor	Dermorphin (30 or 100 pmol)	Rat (SD)	↑ relative ventilator minute volume; ↑respiratory rate; ↓ CO _2_ production	37
AChE inhibitor	Donepezil	0.4 mg/kg, i.v.	Acetylcholinesterase	Morphine (2 mg/kg, i.v.)	Rabbit	↑ Respiratory rate; ↑ respiratory amplitude; ↑ minute phrenic activity; ↓ phrenic nerve apnea threshold PaCO _2_	38
	Donepezil	0.4 mg/kg, i.v.	Acetylcholinesterase	Buprenorphine (0.02 mg/kg, i.v.)	Rabbit	↑ Respiratory rate; ↑ respiratory amplitude; ↑ minute phrenic activity	39
	RA _6_	1 mg i.v., 2 mg s.c.	Acetylcholinesterase	Morphine (8 mg, i.v.)	Rabbit	↑ Respiratory rate; ↓ PaCO _2_	40
	RA _7_	1 or 2 mg, i.v.	Acetylcholinesterase	Morphine (8 mg, i.v.)	Rabbit	↑ Respiratory rate; ↓ PaCO _2_	40
	RA _15_	0.25 or 0.5 mg, i.v.	Acetylcholinesterase	Morphine (8 mg, i.v.)	Rabbit	↑ Respiratory rate; ↓ PaCO _2_	40
	Physostigmine	0.05 or 0.1 mg, i.v.	Acetylcholinesterase	Morphine (8 mg, i.v.)	Rabbit	↓ PaCO _2_	40
Others	*4-aminopyridine*	0.25 mg/kg, i.v.	Potassium channel blocker	Fentanyl (0.6–0.9 mg)	Human	↑ Respiratory rate; ↑ tidal volume; ↑ maximum occlusion pressure; ↓ PaCO _2_	41
	*Glycyl-L-* *glutamine*	1–100 nmol, i.c.v.	Brainstem neurons	Morphine (40 nmol, i.c.v.)	Rat (SD)	Inhibited hypercapnia (PaCO _2_), hypoxia (PaO _2_), and acidosis (blood pH) evoked by morphine	42
	*Thyrotropin-* *releasing* *hormone*	2–5 mg/kg, i.v., i.t.	–	Morphine (5–15 mg/kg, i.v.)	Rat (SD)	↑ Respiratory rate; ↑ tidal volume; ↓ PaCO _2_	43
	*Taltirelin*	1–2 mg/kg, i.v., i.t.	–	Morphine (5–15 mg/kg, i.v.)	Rat (SD)	↑ Respiratory rate; ↑ tidal volume; ↓ PaCO _2_; ↑ PaO _2_	43

5-HT, 5-hydroxytryptamine; α4β2, alpha-4 beta-2 nicotinic receptor; AMPA, α-amino-3-hydroxy-5-methyl-4-isoxazolepropionic acid; D _1_, dopamine receptor D1; GIRK, G-protein-gated inwardly rectifying potassium; i.c.v., intracerebroventricular; i.m., intramuscular; i.p., intraperitoneal; i.t., intrathecal; i.v., intravenous; KM, Kun Ming; NMDA, N-methyl-D-aspartate; PaCO _2_, partial pressure of carbon dioxide; PaO _2_, partial pressure of oxygen; PDE4, phosphodiesterase 4; PKA, protein kinase A; SaO _2_, oxygen saturation; s.c., subcutaneous; SD, Sprague Dawley; WH, Wistar Han.

Ampakines are positive allosteric modulators of the α-amino-3-hydroxy-5-methyl-4-isoxazolepropionic acid (AMPA) receptor, which has a key role in the maintenance of respiratory drive in the pre-Botzinger complex and other central nervous system sites ^2^. In both animals and humans, ampakines stimulate respiratory drive, particularly under hypoventilatory conditions ^2^. CX717 is one of two ampakines tested in humans that have been shown to partially reverse alfentanil-induced respiratory depression ^7^. The other, CX1739, has been assessed in a phase 2 clinical trial for its capacity to antagonize remifentanil-induced respiratory depression; however, the results are not published as yet (ClinicalTrials.gov; Identifier: NCT02735629). Apart from evoking respiratory stimulation, ampakines augment morphine-induced antinociception in rats, showing the utility of combining an opioid with an ampakine to produce potent pain relief but with a superior respiratory safety profile compared with an equi-analgesic dose of morphine alone ^8^. More recently, single intravenous (i.v.) bolus doses of the ampakine LCX001 prevented and reversed fentanyl-induced respiratory depression in rats by strengthening respiratory frequency and minute ventilation whilst maintaining opioid analgesia ^9^. Encouragingly, i.v. LCX001 also produced dose-dependent antinociception in rats ^9^.

In other work, i.v. administration of either nicotine or the α4β2 nicotinic acetylcholine receptor agonist A85380, but not the α7 nicotinic acetylcholine receptor agonist PNU282987, rapidly reversed fentanyl-induced respiratory depression and apnea in rats in a manner comparable to i.v. dosing with the opioid receptor antagonist naloxone ^10^. Additionally, i.v. A85380 potentiated fentanyl-induced antinociception in rats consistent with earlier work showing that agonists of the nicotinic α4β2 receptor evoke antinociception ^10^. Furthermore, A85380 had a modest effect on fentanyl-induced sedation in rats ^10^. Remifentanil is a highly potent respiratory depressant that is particularly difficult to reverse by either a low dose of naloxone or an ampakine in a recent clinical trial ^11^. Thus, the finding that i.v. remifentanil-induced apnea was markedly reduced by co-administration of i.v. A85380 is of particular interest ^10^. The respiratory protective effects of A85380 appear to be underpinned by the fact that the nicotinic acetylcholine receptor subunits α4 and β2 are expressed by the medullary respiratory network and activation of α4β2 receptors increases respiratory rhythm ^10^. Additionally, α4β2 receptors are present in the carotid bodies and so they may also potentially contribute to the respiratory stimulant effects of A85380 ^10^. The water solubility of A85380 like naloxone, together with its much longer half-life at approximately 7 hours compared with 15–30 minutes for naloxone ^10^, support the progression of this compound towards clinical trials.

Doxapram is widely used in veterinary practice to reverse opioid-induced respiratory depression. In goats, i.v. doxapram reduced etorphine-induced respiratory depression by rapid reversal of all respiratory parameters except tidal volume ^12^. In adult humans, doxapram is used to reverse respiratory depression post-anesthesia by direct input on brainstem centers with differential effects on the pre-Botzinger complex and the downstream motor output (XII) ^13^. In preterm infants with apnea of prematurity insensitive to caffeine treatment, doxapram infusion significantly reduced apnea episodes primarily by its effect on respiratory drive rather than on respiratory muscle ^14^. Interestingly, the molecular mechanism underpinning the respiratory stimulant effects of doxapram is restricted to the positive enantiomer and involves inhibition of human TWIK-related acid-sensitive K ^+^-channels (TASK), in particular TASK-1 and TASK-3 channels that are expressed in the carotid body ^15, 16^.

Recent work in anaesthetized rabbits has shed new light on the mechanism by which 5-HT receptor agonists stimulate respiratory parameters, including minute ventilation, respiratory rate, and tidal volume ^17^. Specifically, bilateral microinjection of 5-HT caused excitatory activity of the pre-Botzinger complex via a mechanism mediated by 5-HT _1A_ and 5-HT _3_ receptors ^17^.

Other pharmacological classes assessed for their ability to blunt opioid-induced respiratory depression include PKA inhibitors, GIRK inhibitors, and thyrotropin-releasing hormone (TRH) analogs. Specifically, fentanyl-induced respiratory depression was attenuated in unrestrained rats by intracerebroventricular (i.c.v.) bolus doses of the PKA inhibitor H89 ^36^ and by the GIRK inhibitor tertiapin-Q ^36^. In anaesthetized rats, TRH and its long-acting analog, taltirelin, evoked a marked increase in respiratory rate, tidal volume, and blood oxygenation after i.v. co-administration with morphine ^43^.

In a proof-of-concept clinical study in healthy human subjects, i.v. infusion of the NMDA receptor antagonist esketamine at a subanesthetic dose dose-dependently reversed respiratory depression induced by i.v. remifentanil ^35^. This was underpinned by a stimulatory effect on ventilatory CO _2_ chemosensitivity that was otherwise reduced by remifentanil alone ^35^. The esketamine effect had a rapid onset of action and it was driven by plasma pharmacokinetics ^35^. By contrast, esketamine had little or no effect on resting ventilation. Of concern, however, is that two of 14 subjects withdrew from the study owing to the psychotomimetic side-effects of esketamine ^35^.

## Conclusions

The US opioid epidemic has focused attention on the discovery of respiratory stimulants to reverse opioid-induced respiratory depression whilst sparing opioid analgesia. Although progress has been made, most studies have been confined to the preclinical setting. Very few molecules have entered clinical development, and there are currently no ongoing clinical trials of respiratory stimulants registered on ClinicalTrials.gov (accessed 5 December 2019). Hence, considerable work remains before respiratory stimulant molecules with promising preclinical and/or human data become available for use in clinical practice.
